# A Tentative Mechanism of Solubilization of Neuropathy Target Esterase from Chicken Embryo Brain by Phospholipase A_2_

**DOI:** 10.1100/tsw.2008.51

**Published:** 2008-04-01

**Authors:** Josef Seifert

**Affiliations:** Department of Plant and Environmental Protection Sciences, University of Hawaii, Honolulu, HI 96822, USA

**Keywords:** chicken embryo brain membranes, L-α-lysophosphatidylcholine, lysophospholipase, lysophospholipids, neuropathy target esterase, organophosphate-induced distal neuropathy, phenyl valerate carboxylesterases, phospholipase A_2_

## Abstract

The neuropathy target esterase is a membrane-bound enzyme linked to organophosphate-induced distal neuropathy. Here we report a tentative mechanism of its solubilization from chicken embryo brains by using phospholipase A_2_. The enzyme was released from brain membranes after degradation of their structural phospholipids initiated by phospholipase A_2_. L-α-lysophosphatidylcholine, tested as a representative product of phospholipid hydrolysis, was identified as a new efficient detergent for solubilization of the neuropathy target esterase.

## INTRODUCTION

The neuropathy target esterase (NTE) is a neural, membrane-bound, phenyl valerate carboxylesterase (PVC) linked to etiology of organophosphate-induced distal neuropathy[1,2]. NTE can be solubilized from brain membrane preparations with ionic or nonionic detergents[3,4], but often with significant losses of enzyme activity. In contrast, more than 90% of NTE activity has been recovered from chicken embryo brain membranes by using phospholipase $A_2$ ($PLA_2$)[5]. In the work reported here, we examined the consequences of $PLA_2$ treatment of chicken embryo brain membranes on membrane phospholipids and a possible involvement of lysophospholipids in NTE solubilization.

## MATERIALS AND METHODS

### Preparation of Brain Membrane Fragments (BMF) and NTE Solubilization

Brains of 14- to 16-day-old chicken embryos (white leghorns, Asagi, Honolulu) were homogenized in 50 m*M* Tris-HCl buffer, pH 7.5, containing 1 m*M* EDTA and 0.5 *M* NaCl (1:10, w/v) using Elvehjem-Potter homogenizer. The homogenates were centrifuged sequentially at 10,000 and 100,000×*g*. The resulting pellets were resuspended in 50 m*M* Tris-HCl buffer, pH 7.5, and recovered by the centrifugation at 100,000×*g*; this process was repeated twice prior to BMF treatment with $PLA_2$ or L-α-lysophosphatidylcholine.

NTE solubilization by $PLA_2$ (bee venom $PLA_2$; 5–10 units per equivalent to 100–300 mg of wet brain weight; Sigma Chemicals, U.S.) at 37°C for 20 min followed a previously established protocol[5,6]. The supernatant obtained by centrifugation of the reaction mixture of BMF with $PLA_2$ at 100,000×*g* was considered as an NTE soluble fraction. For NTE solubilization by L-α-lysophosphatidylcholine, BMF (equivalent to 100–160 mg of wet brain weight) suspension in an EDTA-free homogenizing buffer was incubated with 0.001–4 mg/ml of L-α-lysophosphatidylcholine (egg yolk, ~99%, ICN/Sigma, U.S.) at 25°C for 20 min. The mixture was cooled to 0°C and centrifuged at 100,000×*g* for 60 min to obtain a supernatant for the determination of NTE activity.

NTE activity was assayed spectrophotometrically with phenyl valerate (1.1 m*M*) as substrate using a microplate reader. Paraoxon (diethyl 4-nitrophenyl phosphate, 67 μ*M*) and mipafox [N,N'-bis(1-methylethyl)-phosphorodiamidic fluoride, 167 μ*M*] were used as specific inhibitors for defining NTE[1].

### Monitoring BMF Phospholipids by Thin Layer Chromatography (TLC)[7]

$PLA_2$ treated/nontreated BMF and/or the pellets obtained by the centrifugation of BMF treated with L-α-lysophosphatidylcholine were extracted by a methyl alcohol-chloroform mixture (2:1, v/v). Two phases were obtained by centrifugation at 3,000×*g* for 15 min. The chloroform phase was dried by anhydrous $Na_2SO_4$ and concentrated under nitrogen at 20°C. The polar phase was concentrated in the vacuum rotary evaporator at 35°C. The extracts were separated on silica gel plates (200 μm thick, with fluorescent indicator, Fisher, U.S.) using a chloroform-methyl alcohol-water (58:31:4) mixture. Development in the second rectangular direction with chloroform-methyl alcohol-formic acid-water (56:25:6:1) mixture initially was used to confirm the separation pattern obtained by the primary solvent system. The separated BMF components were detected by exposure in sequence to iodine vapors followed by spraying with the ninhydrine reagent, molybdate reagent, and by a char reaction[8]. Phospholipid standards (egg yolk L-α-lysophosphatidylcholine; egg yolk L-α-lysophosphatidylethanolamine; egg yolk L-α-phosphatidylcholine; bovine brain L-α-phosphatidylethanolamine; bovine brain L-α-phosphatidylserine; Sigma, U.S.) were separated in parallel with the samples to identify presumptively the major groups of phospholipids.

### Phospholipid Assay

The total phospholipid content in BMF and/or the phospholipids separated by TLC were assayed as phosphates with ammonium molybdate-hydrazine agent after their digestion with 96% sulfuric acid (30 min at 150°C). $KH_2PO_4$ and/or L-α-phosphatidylcholine were used for a construction of the calibration curves; the assay of L-α-phosphatidylcholine also included its separation by TLC.

## RESULTS AND DISCUSSION

L-α-phosphatidylcholine (55–65%), L-α-phosphatidylethanolamine, and L-α-phosphatidylserine (together 35–45%) were the major BMF phospholipids (Fig. 1). A treatment of BMF with $PLA_2$ resulted in a significant decline of the principle phospholipids (Fig. 1). Lysophosphatidylcholines and lysophosphatidylethanolamines were detected early in BMF treated with $PLA_2$, but both fractions diminished on a prolonged incubation of the reaction mixture (Fig. 1). Their instability and low recovery were probably due to their degradation facilitated by lysophospholipase activity of NTE recently reported by Casida's and Glynn's groups[9,10]. A release of NTE from BMF in solubilization by $PLA_2$[5,6] was the ultimate outcome of membrane disintegration initiated by the changes in their phospholipid composition.

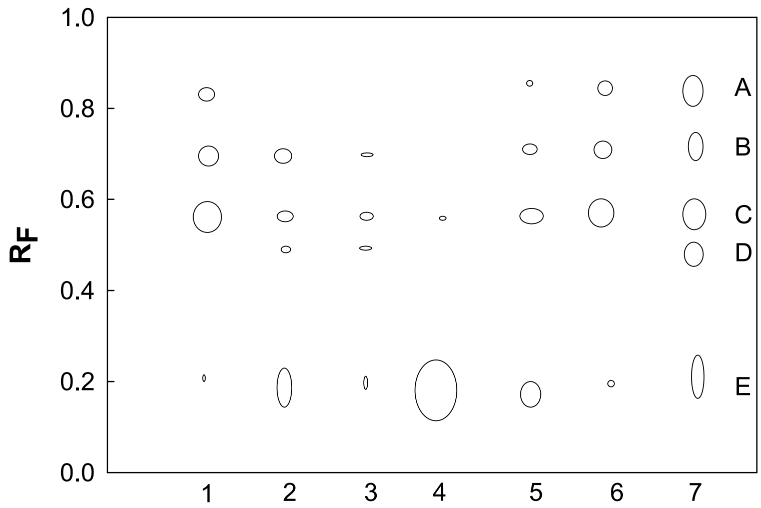

**FIGURE 1.** TLC separation of chicken embryo BMF treated with $PLA_2$ and/or L-α-lysophosphatidylcholine. The schematic presentation is based on analyses of the nonpolar phase of a $CH_3OH$-$CHCl_3$ (2:1, v/v) extract (n independent experiments) of: 1, BMF (n = 12); 2–3, BMF treated with $PLA_2$ (2 – after 15 min, 3 – after 30 min incubation) (n = 5); 4–6, the pellets obtained after 100,000×*g* centrifugation of BMF incubated with varying amounts of L-α-lysophosphatidylcholine (4 – 470 µg/ml; 5 – 47 µg/ml; 6 – 4.7 µg/ml) (n = 2); 7, the standards of L-α-phosphatidylethanolamine (A) (n = 12), L-α-phosphatidylserine (B) (n = 8), L-α-phosphatidylcholine (C) (n = 25), L-α-lysophosphatidylethanolamine (D) (n = 5), and L-α-lysophosphatidylcholine (E) (n = 25). Only a minor amount of lysophosphatidylcholine was detected in the polar phase of the extract of BMF treated with $PLA_2$. The neutral lipids detected by char reaction are not shown ($R_F$ 0.98–1.00).

L-α-lysophosphatidylcholine, selected as a representative product of membrane phospholipid hydrolysis, was an efficient detergent for NTE solubilization (Fig. 2). BMF extraction with L-α-lysophosphatidylcholine at concentrations ≥500 µg/ml yielded 88–100% of the total PVCs, 86–92% of a paraoxon-resistant PVC component, and 78–96% of NTE. The concentrations of L-α-lysophosphatidylcholine that were needed for a complete solubilization of NTE (~$10^{-3}$ *M*) were in the range of its critical mycelial concentrations ($10^{-5}$ – 8 × $10^{-3}$ *M*)[11]. Considering BMF phospholipid content (400–600 µg/ml of BMF preparation), lysophospholipids that would be formed by their complete hydrolysis have the potential to solubilize NTE *in situ*.

Our previous study[6], which revealed BMF as a $PLA_2$ substrate, and the work presented here provide a rationale for the tentative mechanism of NTE solubilization from the chicken embryo BMF by $PLA_2$. The solubilization is initiated by $PLA_2$-catalyzed hydrolysis of BMF phospholipids. A degradation of the structural phospholipids results in NTE release. L-α-lysophosphatidylcholine effectively solubilized NTE, indicating that the lysophospholipids formed from membrane phospholipids may contribute to high yields of the solubilized enzyme. The results of this study also introduce new considerations about the toxic effects of $PLA_2$ as a component of the numerous animal toxins.

## ACKNOWLEDGMENT

The author thanks Dr. J.E. Casida for providing us with mipafox and Dr. B.W. Wilson for his reading and criticism of this manuscript.

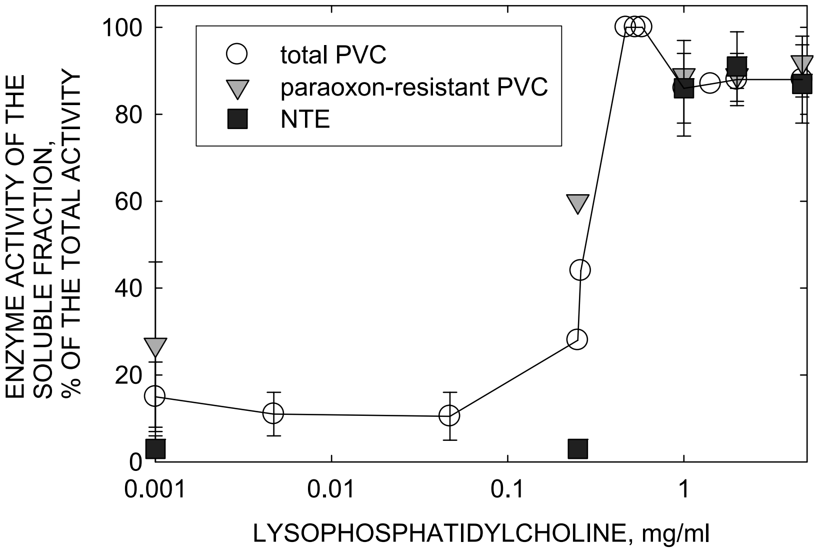

**FIGURE 2.** Solubilization of NTE and other PVCs from chicken embryo BMF by L-α-lysophosphatidylcholine. Enzyme activities are the arithmetic averages of triplicate assays with an error bar for the standard deviation where at least three independent experiments were carried out. 100% activities of the total PVCs, paraoxon-resistant PVCs, and NTE were 450 ± 156, 297 ± 123, and 172 ± 134 pmoles.min$^{-1}$.[mg wet brain]$^{-1}$, respectively.
